# Relative impact of genetic ancestry and neighborhood socioeconomic status on all-cause mortality in self-identified African Americans

**DOI:** 10.1371/journal.pone.0273735

**Published:** 2022-08-29

**Authors:** Hari S. Iyer, Scarlett Lin Gomez, Iona Cheng, Timothy R. Rebbeck

**Affiliations:** 1 Division of Population Sciences, Dana-Farber Cancer Institute, Boston, Massachusetts, United States of America; 2 Department of Epidemiology, Harvard T. H. Chan School of Public Health, Boston, Massachusetts, United States of America; 3 Department of Epidemiology & Biostatistics, University of California, San Francisco, San Francisco, California, United States of America; University of Arizona, UNITED STATES

## Abstract

Self-identified race/ethnicity is a correlate of both genetic ancestry and socioeconomic factors, both of which may contribute to racial disparities in mortality. Investigators often hold *a priori* assumptions, rarely made explicit, regarding the relative importance of these factors. We studied 2,239 self-identified African Americans (SIAA) from the Prostate, Lung, Colorectal and Ovarian screening trial enrolled from 1993–1998 and followed prospectively until 2019 or until death, whichever came first. Percent African genetic ancestry was estimated using the GRAF-Pop distance-based method. A neighborhood socioeconomic status (nSES) index was estimated using census tract measures of income, housing, and employment and linked to participant residence in 2012. We used Directed Acyclic Graphs (DAGs) to represent causal models favoring (1) biomedical and (2) social causes of mortality. Hazard ratios were estimated using Cox models adjusted for sociodemographic, behavioral, and neighborhood covariates guided by each DAG. 901 deaths occurred over 40,767 person-years of follow-up. In unadjusted (biomedical) models, a 10% increase in percent African ancestry was associated with a 7% higher rate of all-cause mortality (HR: 1.07, 95% CI: 1.02, 1.12). This effect was attenuated in covariate adjusted (social) models (aHR: 1.01, 95% CI: 0.96, 1.06). Mortality was lower comparing participants in the highest to lowest nSES quintile following adjustment for covariates and ancestry (aHR: 0.74, 95% CI: 0.57, 0.98, *P*_trend_ = 0.017). Higher African ancestry and lower nSES were associated with higher mortality, but African ancestry was not associated with mortality following covariate adjustment. Socioeconomic factors may be more important drivers of mortality in African Americans.

## Introduction

Racial disparities in health arise from the complex interplay between multiple factors, including structural racism that has generated inequities in societal and institutional factors [1, 2], health care access, individual-level lifestyles and behaviors [3], and genetic factors. These complexities have confounded efforts to narrow these gaps through intervention [4, 5]. Understanding the relative importance of biological and social causes of disparities is critical for proposing effective interventions to reduce these disparities.

Public health scientists favor theories of causation in health disparities research that acknowledge historical policies and societal factors, operating at varying spatiotemporal scales, which shape environmental risk pathways for groups defined by self-identified race or ethnicity (SIRE) [2, 3, 6]. Race/ethnicity is increasingly understood as a multidimensional construct that reflects both how the individual perceives themselves as well as how they are perceived by others [7, 8]. Conceptualizing race/ethnicity as socially assigned is consistent with observations that reported race may change depending on who is performing the classification [9, 10], and that health disparities vary based on phenotypes associated with race, such as gradients in skin color [8, 11]. In addition to social determinants of health, there is ample evidence that disease etiology involves mechanisms occurring at individual, molecular, and cellular levels, which may differ across SIRE groups [12–14]. Improved understanding of the biological variability associated with race and specific health endpoints can offer improvements in clinical care through better targeting of therapies and stratification of risk [15].

Both biomedical and social scientists acknowledge that relying on SIRE or perceived race/ethnicity to infer causality is problematic due to its high correlation with several interrelated biologic and non-biologic pathways that drive disparities [13, 16–18]. Acknowledging the poor specificity of SIRE to understand causes of disparities, scientists have turned to genetic ancestry as a measure of the biological contribution to racial disparities in health [19–21]. Remarkable diversity in genetic ancestry exists, even among members within the same SIRE group [22–24]. This variation may in part explain racial/ethnic differences in disease risk. African ancestry-specific genetic risk variants have been identified for prostate cancer [25], breast cancer [26–28], and numerous other chronic diseases [19]. The frequency and magnitude of effect of risk variants also vary substantially by SIRE [29]. Studies of genetic ancestry and health have historically not controlled for individual and neighborhood variables. In most genetic epidemiologic research, confounding is not considered to be a major threat to study validity because individual-level genetic variation arises through random (Mendelian) assortment [30]. An important exception is population stratification bias, which introduces non-random associations between prevalence of particular alleles and disease in genetic association studies [31].

In this study, we conduct a multilevel analysis to evaluate relative contributions of genetic and sociodemographic correlates of SIRE that may drive disparities using a database containing genetic, lifestyle, behavior, and socioeconomic data at individual and area-level scales [32, 33]. Our first goal is to compare the impacts of genetic ancestry and sociodemographic contextual characteristics on all-cause mortality using data from self-identified African American (SIAA) participants in the Prostate, Lung, Colorectal, and Ovarian (PLCO) cancer screening trial. Our second goal is to provide a framework to guide design and analysis of studies examining the role of ancestry-related biological vulnerability that makes causal assumptions regarding relationships between interrelated social, behavioral, and environmental factors explicit [5]. SIAA were chosen because genetic ancestral admixture and socioeconomic factors have been shown to influence disease risk among members of this group [34–38]. In addition, restricting to a single racial/ethnic group limited potential of confounding through unmeasured correlates of race/ethnicity, ancestry, and socioeconomic factors.

## Materials and methods

### Conceptual frameworks for biological and social causes of mortality

We developed two Directed Acyclic Graphs (DAGs) informed by causal theories from a biomedical perspective and social sciences perspective regarding relationships between genetic ancestry, race/ethnicity, and mortality in a hypothetical population of SIAA in the US. DAGs are visual tools for examining causal pathways between exposures, outcomes, and confounding variables in an epidemiologic setting [39]. Causal relationships between two variables in a DAG are indicated by right flowing arrows. Non-causal paths between two variables are indicated through (1) a shared common cause and (2) a shared common effect that has been conditioned on through covariate adjustment or selection. Further details on use of DAGs for study design and bias assessment are available elsewhere [39, 40]. We reviewed literature on studies of genetic ancestry [19, 23, 24, 41], methodological examinations of race in epidemiologic research [3, 10, 42–45] and racial disparities [46] to inform the causal assumptions in our DAGs. More complex DAGs that incorporate time-varying relationships and additional nodes are possible. Testing assumptions made in these more complex situations would require temporally resolved data collection over the life course, and such databases are rarely available. Our goals in constructing these DAGs were to capture the strongest causal assumptions made by public health researchers, and organize our DAGs based on the most common data elements and designs available to most researchers.

**Fig 1:**
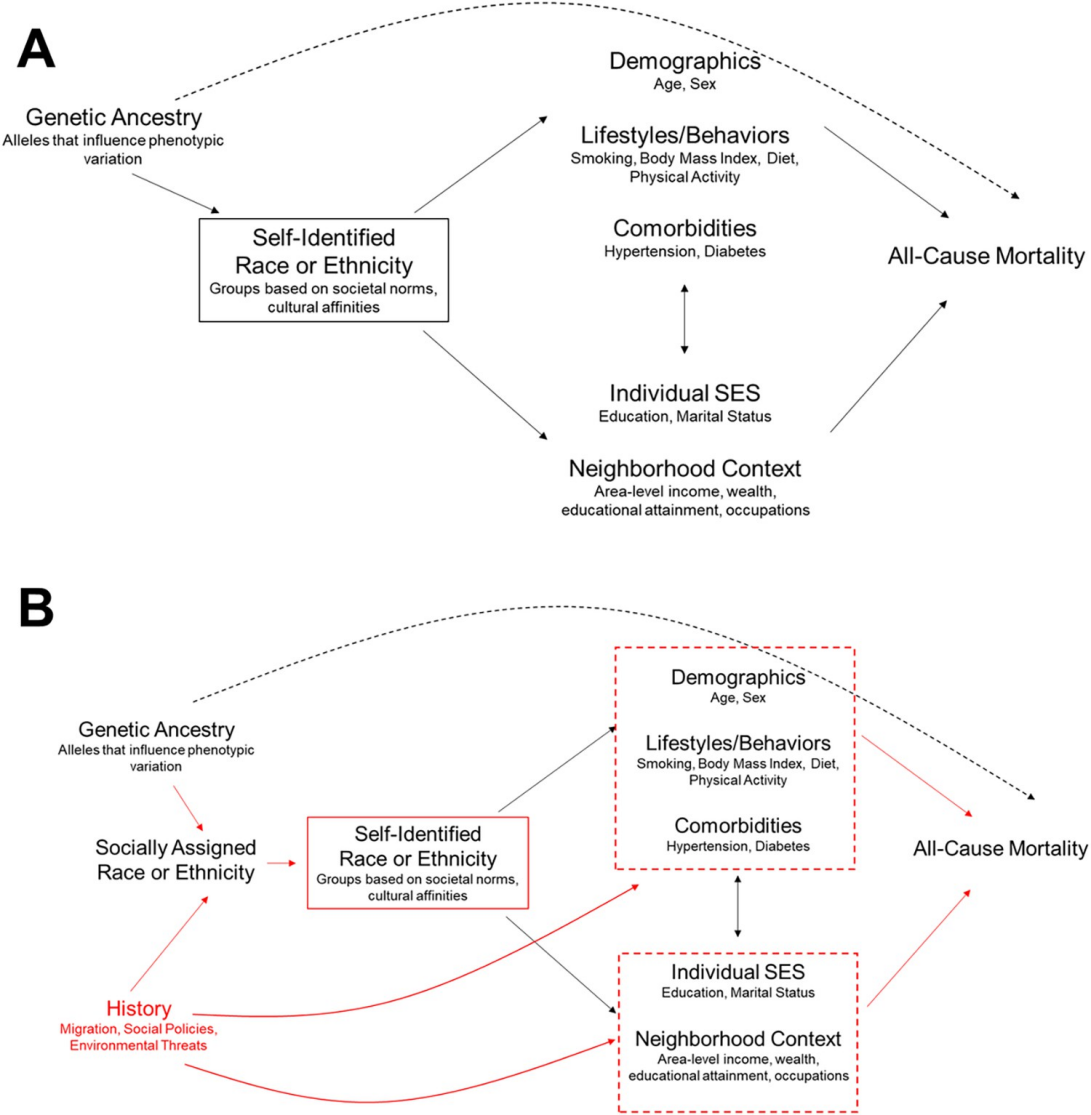
Biomedically-Oriented Causation Framework (A) and Social Science Oriented Causation Framework (B) Applied to Studies Investigating Effects of Genetic Ancestry on Health. Notes: Panel 1A reflects a causal framework favoring a biomedical orientation to causal effects of genetic ancestry (biological correlates of self-identified race or ethnicity (SIRE)) and all-cause mortality. Under this framework, race is considered an individual-level characteristic measured by SIRE. Because genetic ancestry arises from random assortment at conception, and because race/ethnicity is restricted to a single racial/ethnic group, there is no need to adjust for downstream demographic, lifestyle, comorbidities, or SES variables. Panel 1B reflects a causal framework favoring a social sciences orientation to causal effects of genetic ancestry on health. In this conceptualization, socially assigned race is the underlying construct that SIRE is measuring. This framework contains a node for “history”, which captures historical institutional discriminatory practices, such as Jim Crow laws and housing policies that influence racial health disparities in the present. These historical factors are assumed to exert effects on socioeconomic status (via segregation, which concentrates poverty and limits educational and economic opportunities), and demographic and lifestyle factors via psychosocial stress pathways. Selection bias can arise if, through restriction on race/ethnicity, genetic ancestry is correlated with all-cause mortality via demographic and lifestyle factors, as well as socioeconomic factors. Under the assumptions of this social sciences theory of causation, adjusting for these factors can reduce bias through this non-causal pathway, leading to greater validity of findings.

The DAG in Fig 1A illustrates relationships between measured variables under a biomedical theory of disease causation [12, 37]. SIRE reflects individual phenotypes associated with race, as well as lifestyles, cultural practices, sociodemographic, and clinical characteristics [43]. Effects of genetic ancestry are assumed to flow through the race/ethnicity node, which itself drives demographic, lifestyle, comorbidities and SES that ultimately influence risk of death. By restricting the study population to SIAA, any association between genetic ancestry and mortality is assumed to arise through direct effects of genetic ancestry on mortality. Hence, there is no need to adjust for any intermediate variables between SIRE groups and mortality.

Fig 1B displays a DAG informed by a social sciences theory of causation [43, 46, 47]. Here, race/ethnicity is represented by socially assigned race, which is highly correlated with SIRE (the measured variable). Historical factors, such as slavery and Jim Crow segregation laws, which capture impacts of structural racism that continue to influence racial disparities in health in the present [1], are correlated with SIRE. These upstream historical factors drive racial and income segregation, which influences neighborhood socioeconomic status and downstream health behaviors and outcomes by concentrating poverty and limiting educational and economic opportunities [1, 2]. The box around SIRE reflects restriction to only SIAA participants, as before in Fig 1A. Under the assumptions of 1B however, genetic ancestry is correlated with mortality through uncontrolled effects of historical factors and restriction to SIAA, a form of selection bias [40]. This selection bias can be mitigated by adjusting for downstream consequences of historical factors, such as socioeconomic status and other lifestyle and behaviors that arise from living in segregated neighborhoods.

## Study population and design

We constructed a cohort of American-born men and women of African descent who participated in PLCO screening trial. Details about design, eligibility criteria, and outcomes are described in detail elsewhere [48, 49]. Study participants were enrolled from 1993 through 2001 and reported demographic and lifestyle information through questionnaires. Follow-up continued until death or censoring in 2019. Since our focus in this study was to understand the multilevel relationships between neighborhood SES (nSES) and genetic ancestry among SIAA, we restricted the study to 7,843 participants who were classified as SIAA or as having predominantly African ancestry based on principal components analysis using GRAF-pop [50] (n = 135), or SIRE (n = 7,708). Of these, 2,506 had both socioeconomic and ancestry data. After excluding 39 foreign-born participants and 228 participants missing covariate data, 2,239 SIAA participants were retained for analysis. Participants with neither nSES or genetic ancestry had lower educational attainment, were less likely to be married, report history of hypertension and diabetes, and more likely to have withdrawn from the study after follow-up (S1 Table).

The institutional review board at the Dana-Farber Cancer Institute approved the current analysis, which relied on retrospective data collected as part of the Prostate, Lung, Colorectal and Ovarian (PLCO) Cancer Screening Trial. Informed consent was obtained from all participants at baseline.

### Neighborhood socioeconomic status

Residential addresses for PLCO participants were assigned in 2012, following a decision by the original study investigators to include these data after completion of the original PLCO trial [48]. Participants who were deceased or could not be located were geocoded according to their last known address, while those who were still alive were geocoded to their addresses in 2012. Therefore, we assumed that the measured neighborhood contexts are similar to those we would have observed at the start of follow-up for the SIAA study participants. There is some evidence that residential mobility may lead to changes in nSES among cancer patients belonging to different racial/ethnic groups [51]. However, the few studies that have compared analyses of environmental exposures measured at single time points vs residential mobility-weighted measures in relation to agricultural exposures and cancer risk [52] and spatial variability in area-level risk of death in colorectal cancer patients [53] found similar results across exposure assessment approaches.

Neighborhood socioeconomic status (nSES) was assessed using census tract-level measures from the 2000 decennial census and the 2006–2010 American Community Survey. We assigned census tract 2000 measures to those who died prior to 2010, and the American Community Survey measures to those who were alive after 2010.

We conceptualized nSES based on area-level occupational class, income, wealth, and education following constructs proposed by Krieger et al. [54]. Variable selection was guided by the Yost socioeconomic index, developed to study nSES in the California Cancer Registry [55], and using the principal components analysis-based approach of Messer and colleagues [56]. These variables and their constructs are described in S2 and S3 Tables. To construct the nSES measure, we first z-scaled each variable by subtracting the mean and dividing by the standard deviation to normalize the range of values for each measure. We then applied principal components analysis to determine which variables retained loadings of 0.25 or higher on the first principal component [57]. We re-ran principal components analysis on this reduced set of variables (% below poverty level, median home value, % renting homes, median household income, % male managers, % less than high school education, and % total unemployed). The first principal component explained 69.2% of the total variability in component census tract measures and was used as our final nSES score. Correlations between census tract socioeconomic measures used to generate the nSES score are presented in S1 Fig, showing the high clustering of measures reflecting higher social class (median income, home value, men and women in management) and lower social class (poverty, public assistance, empty housing, unemployment).

### Genetic ancestry

Genotype data were obtained from whole blood or buccal samples for 110,562 participants using five different Illumina SNP genotyping arrays: Infinium Global Screening Array (GSA), Oncoarray, Omni25, Omnix, and Omni5 [58]. For participants genotyped on multiple platforms, ancestry was determined by using the genotype data from the Oncoarray platform. Genetic variants were filtered by platform and ancestry, and variants with minor allele frequency less than 1%, variant-level missingness greater than 2%, or Hardy-Weinberg Equilibrium exact p-value <0.001 were removed using PLINK 1.9 [59]. Remaining variants were pruned for linkage equilibrium using PLINK 1.9, using variance inflation factor threshold of 2, and a pairwise r^2^ threshold of 0.2. Heterozygosity outliers were computed and removed at a heterozygosity coefficient F of |F| > 0.2.

Ancestry was estimated using the Genetic Relationship and Fingerprinting (GRAF) statistical method, applied separately for each genotyping platform [50, 60]. Briefly, the GRAF method calculates genetic distances from each subject to three reference populations (European, African, Asian), inferring ancestry and ancestry proportions using a set of “fingerprint” SNPs [60]. Reference classifications rely on study-reported population values from the database of Genotypes and Phenotypes (dbGaP), with European (White, Caucasian, European, European American, and other equivalent terms); African (Black, African, African American, Ghana, Yoruba); Asian (Asian, East Asian, Chinese, Japanese) [50]. Estimates of ancestral proportions (percent) in each of these three reference groups is based on genetic distance scores. Because the contribution of non-African ancestry to admixture was overwhelmingly European among SIAA in our sample, an increase in percent African ancestry is synonymous with a decrease in percent European ancestry.

### Mortality

All-cause mortality was assessed through annual study update questionnaires, reports from relatives, friends or physicians, and linkages with the National Death Index [48]. We investigated cause-specific mortality using ICD-09 codes (cancer: 100; cardiovascular disease: 200–400).

### Statistical analysis

We computed summary statistics using percentages for categorical variables and means (SDs) or medians (interquartile ranges [IQRs]) as appropriate for continuous variables. State of birth was categorized based on census regions. We then examined Kaplan-Meier curves for associations between continuous measures or quintiles of percent African ancestry and nSES across the total population, using age as the time scale. To understand the relationship of genetic ancestry with other covariates, we fit sequentially adjusted Cox proportion hazards models with (1: Unadjusted) no adjustment; (2: Basic) adjustment for age and gender; (3: Multivariable) adjustment for smoking (categories: never smoked cigarettes, current cigarette smoker, former cigarette smoker), marital status (categories: married or living as married, widowed, divorced, separated, never married), education (categories: <8 years, 8–11 years, 12 years or completed high school, post high school training other than college, some college, college graduate, postgraduate), current body mass index (continuous), history of diabetes over follow-up, history of hypertension over follow-up, and % of non-Hispanic Black race/ethnicity in the census tract; (4: Multivariable + Mutual) adjustment for nSES (continuous) because there was no evidence of non-linearity. All covariates were assessed at baseline unless otherwise indicated. We repeated these model fitting procedures with nSES as the primary exposure to compare associations with mortality. African genetic ancestry and nSES were parameterized using continuous (per 10 percentage point increase in African ancestry, 1-unit increase for nSES z-score) and quintiles with a p-value for trend estimated by fitting a model for the median value within each quintile of exposure to assess possible non-linear relationships. Proportional hazards were evaluated by examining plots of Schoenfeld residuals [61]. We used Fine-Gray models for competing risks for analyses of cancer- and cardiovascular-specific mortality [62]. We further examined whether the association between African ancestry and mortality varied by levels of nSES (dichotomized above and below the median) using multiplicative interaction terms. All analyses were performed using R version 4.0.3 and tests were two-sided with alpha = 0.05.

## Results

Baseline study characteristics were similar across samples with complete nSES, complete ancestry, and the final analytic sample (S1 Table). Characteristics of the final analytic sample by quintile of nSES are presented in Table 1. Higher nSES was associated with lower percent African ancestry (mean (SD): quintile 5 (Q5): 69.4% (17.2%) vs quintile 1 (Q1): 77.6% (12.6%), p < .001). Participants with higher nSES were more likely to have been diagnosed with hypertension (Q5: 41.2% vs Q1: 33.9%, p = 0.055). Descriptive characteristics by quintile of African ancestry are reported in S4 Table. Percent African ancestry was associated with lower proportions of postgraduate and college education (p < .001) and lower nSES score (p < .001). We also observed the highest proportion of African ancestry among participants in the Southern Census region.

**Table 1:** Baseline descriptive characteristics of self-identified African American men and women Participating in the prostate, lung, colorectal, and ovarian cancer screening trial by quintiles of neighborhood socioeconomic status, United States, 1993^a^.

	Quintiles of neighborhood Socioeconomic Status						
	Q1	Q2	Q3	Q4	Q5	Overall	*P*
Variable	N = 449	N = 447	N = 447	N = 449	N = 447	N = 2239	
Male (%)	48.8	43.6	42.1	47.7	43.2	45.1	0.17
Age (mean, SD)	61.94 (5.36)	62.24 (5.32)	61.75 (5.23)	61.56 (5.19)	61.22 (4.99)	61.74 (5.23)	0.045
Education (%)							< .001
Less Than 8 Years	2.9	1.6	2	1.6	0.9	1.8	
8–11 Years	18.7	12.1	11.2	6.2	2.9	10.2	
12 Years Or Completed High School	25.2	28.2	17.7	14.7	9.6	19.1	
Post High School Training Other Than College	10.2	13.6	9.2	10.5	7.8	10.3	
Some College	28.3	24.8	29.1	29.8	26	27.6	
College Graduate	7.8	10.7	14.5	14.3	18.8	13.2	
Postgraduate	6.9	8.9	16.3	22.9	34	17.8	
Body Mass Index (mean (SD))	29.45 (5.79)	29.30 (5.85)	29.35 (6.08)	28.82 (5.85)	28.15 (4.69)	29.01 (5.69)	0.003
Marital Status (%)							< .001
Married or Living as Married	47.9	48.8	56.2	55	65.5	54.7	
	16	15.4	11.4	11.1	9.4	12.7	
Divorced	23.8	24.6	25.3	25.2	20.1	23.8	
Separated	4.7	5.6	4.3	4.2	1.3	4	
Never Married	7.6	5.6	2.9	4.5	3.6	4.8	
Smoking Status (%)							0.002
Never Smoked Cigarettes	35	41.6	37.8	39	41.4	38.9	
Current Cigarette Smoker	24.7	19	19.9	16.9	12.8	18.7	
Former Cigarette Smoker	40.3	39.4	42.3	44.1	45.9	42.4	
Census Division (%)							< .001
Northeast	9.1	11	10.3	12.7	13.6	11.3	
South	60.8	55.7	55.3	54.3	60	57.2	
Midwest	29.8	32.7	32.9	29	23.5	29.6	
West	0.2	0.4	0.9	3.6	1.8	1.4	
Other	0	0.2	0.7	0.4	1.1	0.5	
Hypertension over follow-up (%)	33.9	36.5	41.4	41.4	41.2	38.9	0.055
Diabetes over follow-up (%)	12.2	11.6	15	12	15.4	13.3	0.29
nSES score^b^ (mean (SD))	-3.24 (0.95)	-1.49 (0.45)	0.07 (0.43)	1.57 (0.46)	3.26 (0.66)	0.03 (2.35)	< .001
GWAS Ancestry Admixture Percentage							
African (mean (SD))	77.59 (12.63)	76.78 (13.51)	74.06 (13.24)	73.59 (14.27)	69.39 (17.17)	74.28 (14.53)	< .001
European (mean (SD))	20.11 (12.54)	20.96 (13.51)	23.67 (13.25)	24.04 (14.46)	27.83 (16.77)	23.32 (14.43)	< .001
Asian (mean (SD))	2.30 (2.22)	2.26 (2.27)	2.26 (2.22)	2.37 (2.31)	2.78 (6.72)	2.40 (3.62)	0.15
Census tract % African Americans (mean (SD))	91 (12)	81 (25)	69 (32)	37 (34)	29 (32)	61 (37)	< .001

Abbreviations: GWAS, Genome-Wide Association Study Q, quintile, SD, standard deviation.

^a^Characteristics assessed at baseline unless otherwise stated

^b^nSES was assessed at residence in 2012 or at last known residence if deceased

**Fig 2:**
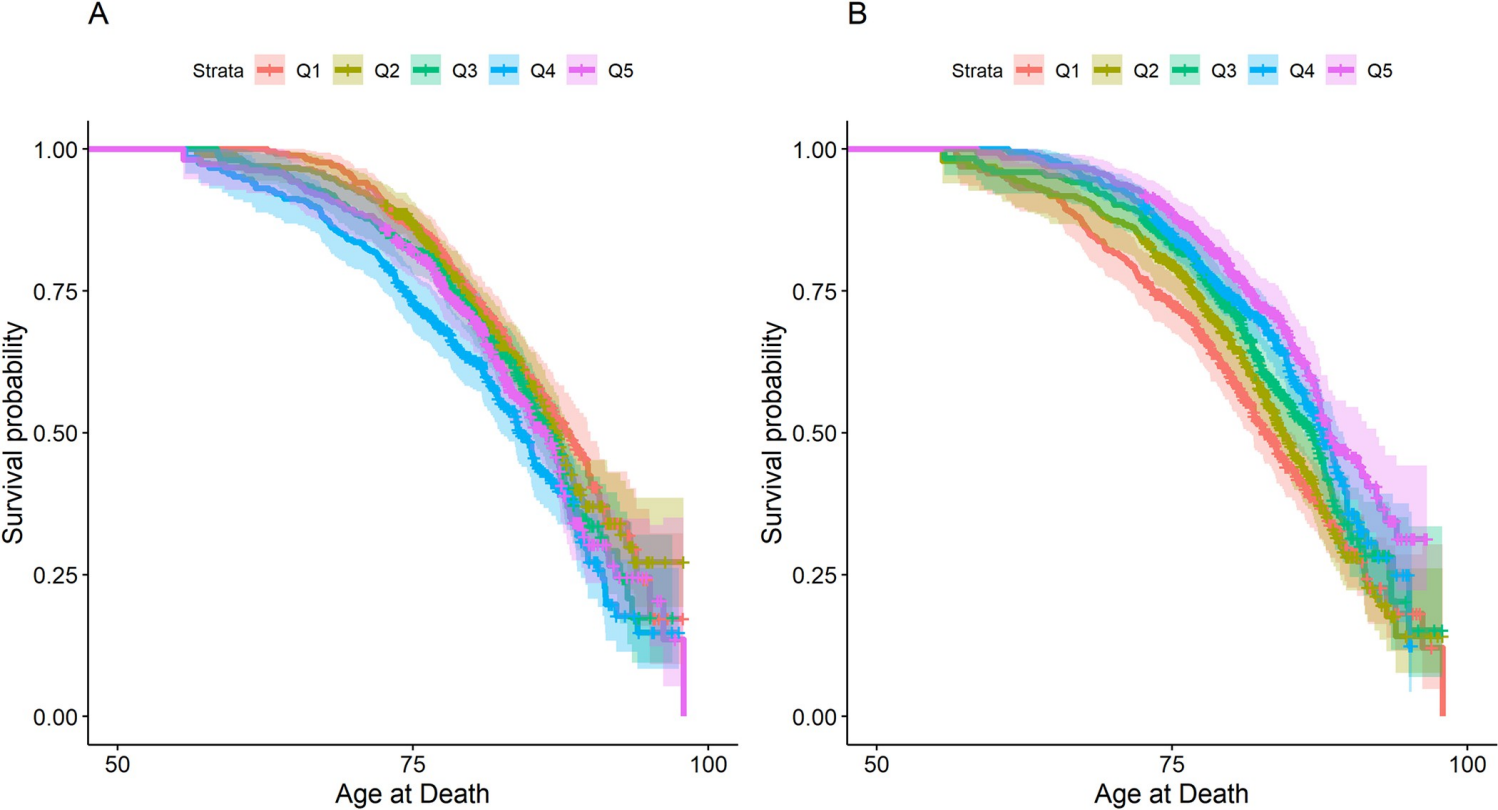
Survival curves for association between quintiles of African Ancestry (panel A) and nSES (panel B) with all-cause mortality among Self-Identified African American Participants in the Prostate, Lung, Colorectal, and Ovarian Cancer Screening Trial, United States, 1993–2019. Abbreviations: nSES, neighborhood Socioeconomic Status, Legend: Log-rank test p-values (Panel A: p = 0.043, Panel B: p < .0001).

There were 901 deaths from any cause over 40,767 person-years of follow-up. In unadjusted survival analysis, higher African ancestry was associated with earlier median age at death (Fig 2A). Higher nSES was associated with later age at death (Fig 2B). For participants in Q1 of percent African ancestry, median age of death was 2 years longer (Ancestry Q1: 88.2 years (95% CI: 86.4, 90.0)) compared to participants in Q5 (86.2 years, 95% CI: 94.1, 87.5, log-rank p-value = 0.043). Participants in Q1 of nSES had a 5.4 year earlier median age of death compared to those in Q5 (nSES Q1: 82.8 years (95% CI: 81.6, 85.1) vs nSES Q5: 88.2 years (95% CI: 87.4, 92.3), log-rank p-value < .0001).

Results from adjusted Cox proportional hazards models for associations between African ancestry, nSES, and all-cause mortality are presented in Table 2. For every 10% increase in African ancestry, there was a 7% increased all-cause mortality (95% CI: 2%, 12%) in unadjusted models. After adjusting for covariates in our multivariable models, the mortality estimate became non-statistically significant with a 2% increase (95% CI: -3%, 7%, *P*_trend_ = 0.41). Adjustment for nSES did not appreciably change results. In contrast, there was a 10% lower mortality rate associated with a 1-unit change in the nSES score (aHR: 0.90, 95% CI: 0.88, 0.93) in unadjusted models. Following covariate adjustment, this association attenuated but remained statistically significant (aHR: 0.94, 95% CI: 0.91, 0.98, *P*_trend_ = 0.015). Further adjustment for percent African ancestry did not change the results (aHR: 0.94, 95% CI: 0.91, 0.98, *P*_trend_ = 0.017). Findings from quintile-based models were consistent with those from linear models.

**Table 2:** Hazard ratios for association between quintile (Q) of African ancestry and neighborhood socioeconomic position (nSES) and mortality among self-identified African American participants in the prostate, lung, colorectal, and ovarian cancer trial, United States, 1993–2019.

	Continuous^a^	Q1 (low)	Q2	Q3	Q4	Q5 (high)	*P*_trend_
**All-Cause Mortality**							
**African Ancestry**							
Deaths		176	172	175	201	177	
Model 1	1.07 (1.02, 1.12)	Referent	1.01 (0.82, 1.25)	1.13 (0.92, 1.40)	1.42 (1.16, 1.73)	1.17 (0.95, 1.44)	0.006
Model 2^b^	1.05 (1.01, 1.10)	Referent	0.99 (0.80, 1.22)	1.10 (0.89, 1.35)	1.33 (1.09, 1.63)	1.13 (0.92, 1.39)	0.024
Model 3^c^	1.02 (0.97, 1.07)	Referent	0.87 (0.70, 1.08)	1.00 (0.81, 1.24)	1.15 (0.93, 1.42)	0.99 (0.79, 1.22)	0.41
Model 4^d^	1.01 (0.96, 1.06)	Referent	0.87 (0.70, 1.07)	1.00 (0.80, 1.23)	1.14 (0.92, 1.41)	0.97 (0.78, 1.20)	0.49
**nSES**							
Deaths		214	206	175	167	139	
Model 1	0.90 (0.88, 0.93)	Referent	0.89 (0.74, 1.08)	0.75 (0.62, 0.92)	0.68 (0.55, 0.83)	0.55 (0.44, 0.68)	< .0001
Model 2^b^	0.90 (0.88, 0.93)	Referent	0.92 (0.76, 1.12)	0.77 (0.63, 0.95)	0.68 (0.55, 0.83)	0.55 (0.44, 0.68)	< .0001
Model 3^c^	0.94 (0.91, 0.98)	Referent	0.99 (0.82, 1.21)	0.85 (0.69, 1.04)	0.80 (0.63, 1.02)	0.75 (0.57, 0.99)	0.015
Model 4^d^	0.94 (0.91, 0.98)	Referent	0.98 (0.81, 1.20)	0.85 (0.69, 1.05)	0.80 (0.63, 1.02)	0.74 (0.57, 0.98)	0.017
**Cancer Mortality**							
**African Ancestry**							
Deaths		54	43	50	63	47	
Model 1	1.02 (0.94, 1.11)	Referent	0.77 (0.52, 1.14)	0.94 (0.64, 1.37)	1.21 (0.85, 1.73)	0.89 (0.61, 1.31)	0.71
Model 2^b^	1.02 (0.94, 1.11)	Referent	0.78 (0.53, 1.16)	0.98 (0.67, 1.42)	1.25 (0.87, 1.79)	0.90 (0.61, 1.33)	0.63
Model 3^c^	1.01 (0.92, 1.10)	Referent	0.73 (0.49, 1.08)	0.96 (0.66, 1.41)	1.17 (0.81, 1.69)	0.87 (0.59, 1.28)	0.8
Model 4^d^	1.02 (0.93, 1.11)	Referent	0.73 (0.49, 1.10)	0.97 (0.66, 1.44)	1.21 (0.83, 1.76)	0.91 (0.61, 1.37)	0.64
**nSES**							
Deaths		55	62	55	44	41	
Model 1	0.94 (0.90, 0.99)	Referent	1.14 (0.8, 1.63)	1.03 (0.71, 1.48)	0.82 (0.55, 1.21)	0.75 (0.51, 1.12)	0.04
Model 2^b^	0.95 (0.90, 1.00)	Referent	1.16 (0.81, 1.65)	1.05 (0.73, 1.51)	0.82 (0.56, 1.22)	0.78 (0.53, 1.16)	0.06
Model 3^c^	1.01 (0.93, 1.08)	Referent	1.30 (0.90, 1.86)	1.26 (0.85, 1.87)	1.11 (0.69, 1.78)	1.18 (0.70, 1.98)	0.6
Model 4^d^	1.01 (0.94, 1.08)	Referent	1.30 (0.90, 1.89)	1.29 (0.87, 1.91)	1.12 (0.70, 1.80)	1.19 (0.71, 2.00)	0.55
**Cardiovascular Disease Mortality**							
**African Ancestry**							
Deaths		68	73	71	82	86	
Model 1	1.10 (1.02, 1.18)	Referent	1.09 (0.79, 1.51)	1.08 (0.78, 1.49)	1.30 (0.95, 1.77)	1.41 (1.04, 1.92)	0.02
Model 2^b^	1.09 (1.01, 1.17)	Referent	1.09 (0.79, 1.50)	1.08 (0.78, 1.49)	1.28 (0.94, 1.75)	1.37 (1.01, 1.86)	0.03
Model 3^c^	1.03 (0.96, 1.11)	Referent	0.98 (0.71, 1.34)	0.94 (0.68, 1.3)	1.04 (0.76, 1.44)	1.12 (0.82, 1.53)	0.48
Model 4^d^	1.03 (0.96, 1.11)	Referent	0.99 (0.71, 1.38)	0.97 (0.69, 1.36)	1.05 (0.75, 1.46)	1.11 (0.8, 1.54)	0.51
**nSES**							
Deaths		102	83	70	72	53	
Model 1	0.91 (0.87, 0.95)	Referent	0.77 (0.58, 1.02)	0.66 (0.49, 0.88)	0.68 (0.50, 0.91)	0.49 (0.35, 0.68)	< .0001
Model 2^b^	0.92 (0.88, 0.95)	Referent	0.79 (0.60, 1.04)	0.68 (0.51, 0.91)	0.69 (0.51, 0.92)	0.51 (0.37, 0.70)	< .0001
Model 3^c^	0.96 (0.91, 1.01)	Referent	0.86 (0.65, 1.14)	0.75 (0.55, 1.02)	0.83 (0.58, 1.18)	0.70 (0.47, 1.03)	0.07
Model 4^d^	0.96 (0.91, 1.02)	Referent	0.85 (0.63, 1.14)	0.76 (0.55, 1.05)	0.84 (0.58, 1.22)	0.72 (0.48, 1.10)	0.12

^a^Per 10 percentage point increase in African ancestry, per 1-unit increase for nSES. Models sequentially adjusted for

^b^age and sex

^c^smoking, marital status, education, Body Mass Index, diabetes, hypertension, and census tract % Non-Hispanic Black residents

^d^mutual adjustment: nSES and African Ancestry. Models 1 and 2 correspond to DAG 1A (biomedical), while Models 3 and 4 correspond to DAG 1B (social sciences).

In cancer-specific mortality models, we found no evidence for associations between either African Ancestry or nSES following multivariable adjustment (Table 2). In models for cardiovascular disease mortality, results were similar to those observed in models for all-cause mortality (Table 2). In unadjusted models, a 10% increase in African ancestry was associated with a 10% increase in cardiovascular-specific mortality (aHR: 1.10, 95% CI: 1.02, 1.18), *P*_trend_ = 0.02, but was attenuated to a non-statistically significant increase following multivariable adjustment. Similarly, there was a statistically significant 9% decrease in cardiovascular-specific mortality (aHR: 0.91, 95% CI: 0.87, 0.95, *P*_trend_ < .0001) in unadjusted models, but this attenuated towards the null following multivariable adjustment (aHR: 0.96, 95% CI: 0.91, 1.01, *P*_trend_ = 0.07).

Results from models for association between African ancestry and mortality stratified by nSES are presented in S5 Table. There was no statistically significant evidence that the association between African ancestry and either all-cause mortality (*P*_het_ = 0.57) or cardiovascular-specific mortality (*P*_het_ = 0.10) varied by nSES. However, among those with low nSES, a 10 percentage point increase in African ancestry was associated with a 14% higher cancer mortality rate (aHR: 1.14, 95% CI: 0.99, 1.32), while among those with high nSES, there was no clear association (aHR: 0.93, 95% CI: 0.83, 1.05, *P*_het_ = 0.025).

## Discussion

After adjustment for individual level covariates and mutual adjustment for SIRE and genetic ancestry, we observed that higher nSES was associated with lower all-cause mortality in SIAA, while no association was observed with genetic ancestry. Covariate adjustment sharply attenuated associations between African ancestry and mortality, while statistically significant associations between nSES and mortality persisted. We found weaker evidence of these patterns for cancer- and cardiovascular-specific mortality, which could be explained in part by smaller numbers of cases. Subsequent mutual adjustment of nSES and genetic ancestry did not appreciably change results.

The unadjusted and minimally adjusted models reflect assumptions regarding relationships between ancestry, covariates and mortality under the biomedical theory of causation (Fig 1A). In these models, we observed a statistically significant increased risk of all-cause mortality with increasing percentage of African ancestry. Despite restricting to SIAA, we observed that decreasing percent African ancestry is associated with higher nSES. Because nSES is associated with both African ancestry and mortality in the data, the biomedical theory erroneously implies that having higher percentage of African ancestry causes lower nSES in adulthood, and therefore higher mortality. This suggests that the causal framework proposed by the biomedical theory in Fig 1A does not adequately reflect relationships between variables observed in our data. In contrast, under assumptions of the social sciences theory of causation (Fig 1B), attenuation of the genetic ancestry-health relationship following covariate adjustment is expected, a result of correctly controlling for factors correlated with ancestry through historically influenced events. Covariate adjustment for individual-level socioeconomic factors mitigates bias and attenuates the association between genetic ancestry and mortality. When adopting a social sciences theory of health disparities, scientists estimating causal effects of genetic ancestry on health outcomes should control for variables assumed to be consequences of historical systematic racism, discrimination, and segregation to avoid conflating genetic ancestry effects with these other correlates of SIRE.

A growing number of multilevel studies have examined African ancestry as a predictor of chronic disease endpoints [20, 21, 36, 46, 63–67]. Geographic analyses of migration patterns in the US have shown that genetic admixture patterns in present-day African Americans are associated with forced movements of people that occurred during the trans-Atlantic slave trade [23, 24]. Descriptive studies of African admixture in the US report marked variability in percentage African ancestry by geographic regions, with the highest levels of African genetic ancestry observed in rural southern states [23]. In most studies, adjusting for socioeconomic and lifestyle variables attenuates the relationship between African ancestry to non-significance [36, 46, 63, 66]. For example, Non et al. reported no evidence of an association between West African ancestry and hypertension following adjustment for education in a study of SIAA [36]. Rao et al. examined associations between West African ancestry and heart disease risk factors within a clinical trial of disease treatments among SIAA, and found limited evidence for a genetic contribution of West African ancestry to heart disease risk [66]. The consistent attenuation of associations between genetic ancestry and health following adjustment for socioeconomic variables within SIAA suggests that socioeconomic and environmental correlates of SIRE, rather than genetic ancestry, are more likely to explain racial disparities in mortality. This evidence favors the social sciences theory of causation, in which historical policies of racial discrimination influence effects of SIRE on health through segregation [2, 68, 69].

Our study has some important limitations. Our measures of nSES may not have been assessed during an etiologically mACKNeaningful period. However, empirical studies examining time-varying measures of neighborhood socioeconomic status have shown that measures over time are highly correlated [51]. Our analyses assume that we have accounted for major confounding variables, and that residual confounding is unlikely to explain our findings. We did not include explicit measures of historic racial discrimination or structural racism because these measures were not available in the PLCO database. We assumed that measured sociodemographic, clinical, and lifestyle variables would be sufficient to control partially for non-genetic correlates of SIAA and mortality. However, social epidemiologists have proposed measures of structural racism that could be linked in future studies [70, 71]. It is possible that the magnitude of associations between nSES and mortality would be attenuated with further adjustment for diet, psychosocial factors, and perceived discrimination. However, these factors could also be considered as mediators of the association between nSES and mortality. Our measure of genetic ancestry may not adequately capture effects of specific ancestry-related mortality variants or related biological pathways. Since participants were recruited as part of a clinical trial, characteristics may not be generalizable to all SIAA. Three of the PLCO sites (Michigan, Alabama, Pennsylvania) implemented dedicated programs to increase participation of Black men and women [72]. These sites were generally more successful in achieving a demographic composition similar to that of their catchment [73]. However, SIAA recruited in PLCO had higher educational attainment and were less likely to smoke, and more likely to exercise compared to the general population of SIAA [73]. Marital status, body mass index, and medical histories were similar. Strengths of the study include a relatively large nation-wide sample, the ability to study multilevel risk factors, and ability to restrict to U.S.-born SIAA for whom the role of historical discrimination is particularly important when assessing associations between ancestry and health.

## Conclusion

In summary, our analysis supports adoption of a social sciences theory of mortality causation when studying effects of genetic ancestry and health in SIAA. The inferences made here do not mean that biological variability within SIRE groups is irrelevant to health but suggest that social and environmental factors may explain a greater proportion of mortality disparities by SIRE than genetic ancestry. We recommend that future large-scale studies of the health effects of genetic ancestry apply similar frameworks that can clarify the interrelationships between important behavioral, social, and environmental correlates of SIRE.

## Supporting information

S1 Fig Correlation matrix for census tract neighborhood socioeconomic variables used to generate neighborhood socioeconomic status score, United States, 1993. Key: pop = Population, mdhval = Median Home Value, mdinc = Median Income, pct_mgr_fem = % of Female Managers, pct_mgr_male = % of Male Managers, lths = % with less than high school education, femhh = %e of households with female head, pubasst = % of residents receiving public assistance, belowpov = % of residents below poverty level, pctvac = % of housing units vacant, pctmunemp = % male unemployment, unemp = % unemployment, crowding = % of crowding, nocar = % with no car, pctrent = % of renter occupied housing, sameres5yrs = % living in same residence for 5 years, res65 = % of residents 65+, femlab = % of females not in labor force, mlab = % of males not in labor force.
(DOCX)

S1 Table Comparison of baseline characteristics of participants with complete information on neighborhood socioeconomic status (n = 3921), African ancestry (n = 4302), those with neither exposure (n = 1372), and those included in final analytic sample (n = 2239), United States, 1993. Abbreviations: GWAS, Genome-Wide Association Study, nSES, neighborhood Socioeconomic Status, Q, quintile, SD, standard deviation.
(DOCX)

S2 Table Variables from the United States 2000 Decennial Census and the American community survey 2006–2010. ^a^From Krieger et al. 1997 [54].
(DOCX)

S3 Table Population characteristics for neighborhood socioeconomic status (nSES) quintiles by principal component analysis for census data (n = 1,135 tracts) among self-identified African American participants in the prostate, lung, colorectal and ovarian cancer screening trial, United States, 1993. Abbreviations: nSES, neighborhood Socioeconomic Status, Note: Residential addresses reflect residence in 2012, or at last known date of contact in 2012.
(DOCX)

S4 Table Baseline descriptive characteristics of self-identified African American participants in the prostate, lung, colorectal, and ovarian cancer screening trial by quintiles of African Ancestry, United States, 1993. Abbreviations: GWAS, Genome-Wide Association Study Q, quintile, SD, standard deviation. ^a^Characteristics assessed at baseline unless otherwise stated; ^b^nSES was assessed at residence in 2012 or at last known residence if deceased.
(DOCX)

S5 Table Hazard ratios for association between African Ancestry and all-cause mortality among self-identified African American participants by levels of nSES in the prostate, lung, colorectal, and ovarian cancer trial, United States, 1993–2019. ^a^Per 10 percentage point increase in African ancestry. Models adjusted for age, sex, smoking, marital status, education, Body Mass Index, diabetes, hypertension, and census tract % Non-Hispanic Black residents.
(DOCX)
