# Supplementary material for: Relative impact of genetic ancestry and neighborhood socioeconomic status on all-cause mortality in self-identified African Americans
Source: PLoS One. 2022 Aug 29;17(8):e0273735. doi: 10.1371/journal.pone.0273735 (PMC9423617; doi:10.1371/journal.pone.0273735)
Supplement: S1 Fig — Key: pop = Population, mdhval = Median Home Value, mdinc = Median Income, pct_mgr_fem = % of Female Managers, pct_mgr_male = % of Male Managers, lths = % with less than high school education, femhh = %e of households with female head, pubasst = % of residents receiving public assistance, belowpov = % of residents below poverty level, pctvac = % of housing units vacant, pctmunemp = % male unemployment, unemp = % unemployment, crowding = % of crowding, nocar = % with no car, pctrent = % of renter occupied housing, sameres5yrs = % living in same residence for 5 years, res65 = % of residents 65+, femlab = % of females not in labor force, mlab = % of males not in labor force. (DOCX) [file pone.0273735.s001.docx]

**S1 Fig. Correlation Matrix for Census Tract Neighborhood Socioeconomic Variables Used to Generate neighborhood socioeconomic status score, United States, 1993**


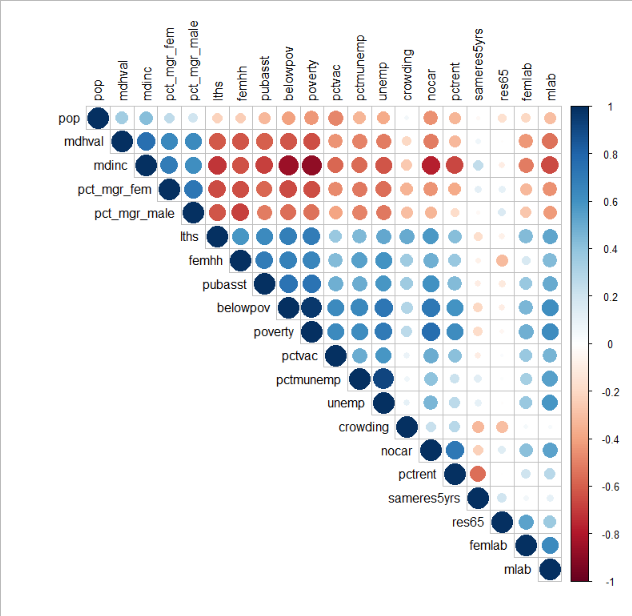


Key: pop=Population, mdhval=Median Home Value, mdinc=Median Income, pct_mgr_fem=% of Female Managers, pct_mgr_male=% of Male Managers, lths=% with less than high school education, femhh=%e of households with female head, pubasst=% of residents receiving public assistance, belowpov=% of residents below poverty level, pctvac=% of housing units vacant, pctmunemp=% male unemployment, unemp=% unemployment, crowding=% of crowding, nocar=% with no car, pctrent=% of renter occupied housing, sameres5yrs=% living in same residence for 5 years, res65=% of residents 65+, femlab=% of females not in labor force, mlab=% of males not in labor force
