# Supplementary material for: Relative impact of genetic ancestry and neighborhood socioeconomic status on all-cause mortality in self-identified African Americans
Source: PLoS One. 2022 Aug 29;17(8):e0273735. doi: 10.1371/journal.pone.0273735 (PMC9423617; doi:10.1371/journal.pone.0273735)
Supplement: S1 Table — Abbreviations: GWAS, Genome-Wide Association Study, nSES, neighborhood Socioeconomic Status, Q, quintile, SD, standard deviation. (DOCX) [file pone.0273735.s002.docx]

**S1 Table. Comparison of baseline characteristics of participants with complete information on neighborhood socioeconomic status (n=3921), African Ancestry (n=4302), those with neither exposure (n=1372), and those included in final analytic sample (n=2239), United States, 1993**

|  |  | | **Sample contains** | | |
| --- | --- | --- | --- | --- | --- |
|  | **nSES** | **African Ancestry** | | **Neither** | **Both (final analytic sample)** |
| **n** | **3921** | **4302** | | **1372** | **2239** |
| Male (%) | 46.3 | 41.7 | | 43.5 | 45.1 |
| Age (mean, SD) | 62.51 (5.37) | 61.84 (5.33) | | 62.46 (5.48) | 61.74 (5.23) |
| Education (%) |  |  | |  |  |
| Less Than 8 Years | 2.7 | 3.3 | | 7 | 1.8 |
| 8-11 Years | 13.6 | 12.5 | | 19 | 10.2 |
| 12 Years Or Completed High School | 19.8 | 21 | | 20.8 | 19.1 |
| Post High School Training Other Than College | 10.4 | 10.6 | | 9.9 | 10.3 |
| Some College | 27.4 | 24.9 | | 20.9 | 27.6 |
| College Graduate | 11.7 | 12 | | 10.5 | 13.2 |
| Postgraduate | 14.4 | 15.6 | | 11.8 | 17.8 |
| Current Body Mass Index (mean (SD)) | 28.98 (5.94) | 29.48 (5.80) | | 29.60 (5.91) | 29.01 (5.69) |
| Marital Status (%) |  |  | |  |  |
| Married or Living as Married | 52.6 | 52.1 | | 46.1 | 54.7 |
| Widowed | 14.7 | 15.2 | | 17 | 12.7 |
| Divorced | 22.6 | 23 | | 25.2 | 23.8 |
| Separated | 4.7 | 4.5 | | 6.2 | 4 |
| Never Married | 5.3 | 5.1 | | 5.4 | 4.8 |
| Smoking Status (%) |  |  | |  |  |
| Never Smoked Cigarettes | 37.7 | 41.5 | | 43.2 | 38.9 |
| Current Cigarette Smoker | 20.9 | 17.4 | | 19.4 | 18.7 |
| Former Cigarette Smoker | 41.4 | 41.2 | | 37.4 | 42.4 |
| Census Division (%) |  |  | |  |  |
| Missing | 0.1 | 0.1 | | 0.2 | 0 |
| Northeast | 10.8 | 9.2 | | 8.7 | 11.3 |
| South | 57.5 | 67.8 | | 71.4 | 57.2 |
| Midwest | 30.2 | 21.6 | | 18.8 | 29.6 |
| West | 1 | 1.1 | | 1 | 1.4 |
| Other | 0.4 | 0.3 | | 0 | 0.5 |
| Hypertension (%) | 30.2 | 36.5 | | 15.7 | 38.9 |
| Diabetes (%) | 10.8 | 13.5 | | 6.2 | 13.3 |
| Withdrew over follow-up (%) | 0.3 | 0.8 | | 14.9 | 0.1 |
| nSES score (mean (SD)) | 0.03 (2.25) |  | |  | 0.03 (2.35) |
| GWAS Ancestry Admixture Percentage |  |  | |  |  |
| African (mean (SD)) |  | 76.37 (14.08) | |  | 74.28 (14.53) |
| European(mean (SD)) |  | 21.35 (14.03) | |  | 23.32 (14.43) |
| Asian (mean (SD)) |  | 2.28 (3.03) | |  | 2.40 (3.62) |
| Census tract % African American (mean (SD)) | 66 (36) |  | |  | 61 (37) |

Abbreviations: GWAS, Genome-Wide Association Study, nSES, neighborhood Socioeconomic Status, Q, quintile, SD, standard deviation
