# Supplementary material for: Relative impact of genetic ancestry and neighborhood socioeconomic status on all-cause mortality in self-identified African Americans
Source: PLoS One. 2022 Aug 29;17(8):e0273735. doi: 10.1371/journal.pone.0273735 (PMC9423617; doi:10.1371/journal.pone.0273735)
Supplement: S2 Table — aFrom Krieger et al. 1997 [54]. (DOCX) [file pone.0273735.s003.docx]

**S2 Table. Variables from the United States 2000 Decennial Census and the American Community Survey 2006-2010**

| **Variable** | **Construct^a^** | **Census 2000** | **ACS 2006-2010** |
| --- | --- | --- | --- |
| Percent with no car | Assets/wealth | x | x |
| Percent on public assistance | Presence of poverty | x | x |
| Percent below poverty | Presence of poverty | x | x |
| Percent of housing units vacant | Assets/wealth | x | x |
| Percent of crowding | Crowding: at household level, more people than rooms | x | x |
| Percent of female head of households with children | Class | x | x |
| Percent of residents 65 and older | Class | x | x |
| Percent living in the same residence for 5 years or more | Class | x | x |
| Percent of Unemployed Men | Unemployment: persons actively seeking employment | x | x |
| Percent of females not in labor force | Working class: nonsupervisory employees | x | x |
| Percent of men not in labor force | Working class: nonsupervisory employees | x | x |
| Percent of poverty | Presence of poverty | x | x |
| Median value for all owner occupied housing units | Assets/wealth | x | x |
| Percent of renter occupied housing units | Assets/wealth | x | x |
| Median Household Income | Income | x | x |
| Percent of males in management occupations | Class: supervisory employees | x | x |
| Percent of females in management occupations | Class: supervisory employees | x | x |
| Total Population | Urbanicity | x | x |
| Percent less than a high school degree | Educational attainment | x | x |
| Percent of unemployed | Unemployment: persons actively seeking employment | x | x |

^a^From Krieger et al. 1997 [54]
