# Supplementary material for: Relative impact of genetic ancestry and neighborhood socioeconomic status on all-cause mortality in self-identified African Americans
Source: PLoS One. 2022 Aug 29;17(8):e0273735. doi: 10.1371/journal.pone.0273735 (PMC9423617; doi:10.1371/journal.pone.0273735)
Supplement: S3 Table — Abbreviations: nSES, neighborhood Socioeconomic Status, Note: Residential addresses reflect residence in 2012, or at last known date of contact in 2012. (DOCX) [file pone.0273735.s004.docx]

**S3 Table. Population characteristics for neighborhood socioeconomic status (nSES) quintiles by principal component analysis for census data (n=1,135 tracts) among Self-identified African American participants in the Prostate, Lung, Colorectal and Ovarian Cancer screening trial, United States, 1993**

|  | **Quintiles of nSES** | | | | |  |
| --- | --- | --- | --- | --- | --- | --- |
| **Census tract characteristics (mean (SD))** | **Q1** | **Q2** | **Q3** | **Q4** | **Q5** | **Total** |
| % on public assistance | 14 (5) | 8 (4) | 5 (2) | 2 (2) | 1 (1) | 6 (5) |
| % below poverty level | 40 (9) | 29 (7) | 18 (6) | 11 (6) | 5 (4) | 21 (14) |
| % female heads of household with children | 19 (5) | 16 (4) | 12 (4) | 8 (3) | 5 (2) | 12 (7) |
| Median home value, 1000 USD | 62.9 (40) | 82.7 (42.2) | 117.2 (61) | 172.4 (80.9) | 326.8 (158.6) | 152.4 (129.3) |
| Median income, 1000 USD | 22.7 (5.7) | 30.4 (5.6) | 38.3 (7.9) | 50.9 (11.3) | 85.4 (25.6) | 45.5 (25.8) |
| % of females in management occupations | 19 (9) | 27 (8) | 33 (9) | 41 (10) | 54 (11) | 35 (15) |
| % adults with less than high school degree | 28 (9) | 22 (7) | 17 (7) | 12 (5) | 6 (4) | 17 (10) |
| % unemployed | 26 (9) | 20 (7) | 14 (7) | 9 (4) | 6 (4) | 15 (10) |

Abbreviations: nSES, neighborhood Socioeconomic Status, Note: Residential addresses reflect residence in 2012, or at last known date of contact in 2012.
