# Supplementary material for: Relative impact of genetic ancestry and neighborhood socioeconomic status on all-cause mortality in self-identified African Americans
Source: PLoS One. 2022 Aug 29;17(8):e0273735. doi: 10.1371/journal.pone.0273735 (PMC9423617; doi:10.1371/journal.pone.0273735)
Supplement: S4 Table — Abbreviations: GWAS, Genome-Wide Association Study Q, quintile, SD, standard deviation. aCharacteristics assessed at baseline unless otherwise stated; bnSES was assessed at residence in 2012 or at last known residence if deceased. (DOCX) [file pone.0273735.s005.docx]

**S4 Table. Baseline Descriptive Characteristics of Self-identified African American Participants in the Prostate, Lung, Colorectal, and Ovarian Cancer Screening Trial by Quintiles of African Ancestry^a^, United States, 1993**

|  | **Quintiles of African Ancestry** | | | | |  |  |
| --- | --- | --- | --- | --- | --- | --- | --- |
|  | **Q1** | **Q2** | **Q3** | **Q4** | **Q5** | **Overall** | ***P*** |
| **n** | **453** | **463** | **441** | **445** | **437** | **2239** |  |
| Male (%) | 41.7 | 42.5 | 45.8 | 47.6 | 47.8 | 45.1 | 0.21 |
| Age (mean, SD) | 62.4 (5.31) | 62 (5.45) | 61.29 (4.92) | 61.22 (5.07) | 61.78 (5.29) | 61.74 (5.23) | 0.003 |
| Education (%) |  |  |  |  |  |  | <.001 |
| Less Than 8 Years | 0.9 | 1.1 | 1.6 | 1.6 | 3.9 | 1.8 |  |
| 8-11 Years | 6.2 | 8.2 | 10.4 | 14.2 | 12.4 | 10.2 |  |
| 12 Years Or Completed High School | 15 | 17.3 | 20.2 | 22.2 | 20.8 | 19.1 |  |
| Post High School Training Other Than College | 7.9 | 11 | 11.1 | 11.9 | 9.4 | 10.3 |  |
| Some College | 26 | 28.7 | 27 | 25.4 | 30.9 | 27.6 |  |
| College Graduate | 16.1 | 14.3 | 11.6 | 13 | 11 | 13.2 |  |
| Postgraduate | 27.8 | 19.4 | 18.1 | 11.7 | 11.7 | 17.8 |  |
| Current Body Mass Index (mean (SD)) | 28.39 (5.27) | 29 (5.91) | 28.66 (5.22) | 29.62 (5.89) | 29.41 (6.06) | 29.01 (5.69) | 0.007 |
| Marital Status (%) |  |  |  |  |  |  | 0.074 |
| Married or Living as Married | 59.4 | 50.3 | 57.8 | 51.5 | 54.5 | 54.7 |  |
| Widowed | 11.3 | 14.9 | 9.5 | 13 | 14.6 | 12.7 |  |
| Divorced | 23 | 23.8 | 25.9 | 24.9 | 21.5 | 23.8 |  |
| Separated | 2.9 | 4.5 | 3.4 | 5.2 | 4.1 | 4 |  |
| Never Married | 3.5 | 6.5 | 3.4 | 5.4 | 5.3 | 4.8 |  |
| Smoking Status (%) |  |  |  |  |  |  | 0.56 |
| Never Smoked Cigarettes | 35.8 | 39.3 | 38.8 | 38.4 | 42.6 | 38.9 |  |
| Current Cigarette Smoker | 18.3 | 20.3 | 19 | 19.3 | 16.2 | 18.7 |  |
| Former Cigarette Smoker | 45.9 | 40.4 | 42.2 | 42.2 | 41.2 | 42.4 |  |
| Census Division (%) |  |  |  |  |  |  | <.001 |
| Northeast | 15.7 | 12.3 | 9.8 | 11.9 | 6.9 | 11.3 |  |
| South | 48.3 | 52.5 | 58.7 | 57.5 | 69.6 | 57.2 |  |
| Midwest | 32.2 | 32.2 | 30.4 | 29.9 | 22.9 | 29.6 |  |
| West | 2 | 2.8 | 0.9 | 0.7 | 0.5 | 1.4 |  |
| Other | 1.8 | 0.2 | 0.2 | 0 | 0.2 | 0.5 |  |
| Hypertension (%) | 39.1 | 39.7 | 41.3 | 36.6 | 37.5 | 38.9 | 0.65 |
| Diabetes (%) | 13.7 | 12.5 | 14.3 | 13 | 12.8 | 13.3 | 0.94 |
| nSES score^b^ (mean (SD)) | 0.83 (2.3) | 0.09 (2.28) | -0.1 (2.31) | -0.28 (2.3) | -0.41 (2.39) | 0.03 (2.35) | <.001 |
| GWAS Ancestry Admixture Percentage |  |  |  |  |  |  |  |
| African (mean (SD)) | 51.42 (10) | 68.91 (2.87) | 77.28 (1.98) | 83.76 (1.91) | 91 (2.93) | 74.28 (14.53) | <.001 |
| European(mean (SD)) | 45.73 (10.28) | 28.56 (3.91) | 20.36 (3.05) | 13.91 (2.92) | 7.1 (3.27) | 23.32 (14.43) | <.001 |
| Asian (mean (SD)) | 2.85 (6.67) | 2.53 (2.45) | 2.36 (2.32) | 2.32 (2.18) | 1.89 (2.02) | 2.40 (3.62) | 0.002 |
| Census tract % African American (mean (SD)) | 54 (38) | 60 (38) | 64 (36) | 64 (37) | 66 (36) | 61 (37) | <.001 |

Abbreviations: GWAS, Genome-Wide Association Study Q, quintile, SD, standard deviation. ^a^Characteristics assessed at baseline unless otherwise stated; ^b^nSES was assessed at residence in 2012 or at last known residence if deceased
