# Supplementary material for: Relative impact of genetic ancestry and neighborhood socioeconomic status on all-cause mortality in self-identified African Americans
Source: PLoS One. 2022 Aug 29;17(8):e0273735. doi: 10.1371/journal.pone.0273735 (PMC9423617; doi:10.1371/journal.pone.0273735)
Supplement: S5 Table — aPer 10 percentage point increase in African ancestry. Models adjusted for age, sex, smoking, marital status, education, Body Mass Index, diabetes, hypertension, and census tract % Non-Hispanic Black residents. (DOCX) [file pone.0273735.s006.docx]

**S5 Table.** **Hazard Ratios for Association between African Ancestry and all-cause mortality among Self-Identified African American participants by levels of nSES in the Prostate, Lung, Colorectal, and Ovarian Cancer Trial, United States, 1993-2019**

| **Continuous African ancestry^a^** | **Low nSES**  **(range: -11.9, 0.11)** | **High nSES**  **(range: 0.11, 5.41)** | ***P*_het_** |
| --- | --- | --- | --- |
|  | **aHR (95% CI)** | **aHR (95% CI)** |  |
| **All-Cause Mortality** | 1.03 (0.96, 1.10) | 1.00 (0.94, 1.07) | 0.57 |
| **Cancer Mortality** | 1.14 (0.99, 1.32) | 0.93 (0.83, 1.05) | 0.025 |
| **Cardiovascular Disease Mortality** | 0.97 (0.88, 1.08) | 1.10 (0.99, 1.23) | 0.11 |

^a^Per 10 percentage point increase in African ancestry. Models adjusted for age, sex, smoking, marital status, education, Body Mass Index, diabetes, hypertension, and census tract % Non-Hispanic Black residents.
